# Can the count and percentage of immature granulocytes be used to detect disease activity in patients with pemphigus?: A preliminary study

**DOI:** 10.1097/MD.0000000000041747

**Published:** 2025-03-07

**Authors:** Mine Müjde Kuş, Ozan Can Yaray, Perihan Öztürk, Mehmet Kamil Mülayim

**Affiliations:** a Department of Dermatology, Kahramanmaras Sutcu Imam University, Kahramanmaras, Turkey.

**Keywords:** disease severity, Dsg1, Dsg3, immature granulocyte, pemphigus

## Abstract

Recently, immature granulocyte count (IGC) in an automated blood cell counter was introduced as a new inflammatory marker. We aimed to evaluate the relationship between the IGC and percentage of immature granulocyte (IG%) and disease activity in patients with pemphigus. Pemphigus disease area index (PDAI), IGC, IG%, C-reactive protein, neutrophil/lymphocyte ratios, platelet-to-lymphocyte ratio (PLR), anti-desmoglein 1, and anti-desmoglein 3 levels in patients with pemphigus were recorded retrospectively, and the statistical relationship between them was evaluated. Repetitive intra-patient blood samples (74 blood samples) and PDAIs (74 PDAI scores) of 24 patients (12 men, 12 women; mean age 56.30 ± 16.47 years) were included in the study. There was no correlation between PDAI and anti-desmoglein 1, anti-desmoglein 3 levels, and neutrophil/lymphocyte ratio (*r* = 0.040, *P* = .737; *r* = 0.007, *P* = .952; *r* = 0.224, *P* = .055, respectively). A statistically low positive correlation was detected between PDAI and C-reactive protein (*r* = 0.243, *P* = .037). There was a moderate positive correlation between PDAI and IGC, IG%, and PLR (*r* = 0.435, *P* < .001; *r* = 0.412, *P* < .001; *r* = 0.376, *P* = .001, respectively). The effects of independent variables in predicting PDAI were evaluated using multiple linear regression analysis. Accordingly, the cutoff value of the IGC and PLR positively significantly affect the PDAI score (*P* < .05). A 1-unit increase in PLR indicates a 0.009 times increase in PDAI, and a 1-unit increase exceeding the IG cutoff value indicates a 2.836 times increase in PDAI. The IGC and IG% can be used to evaluate pemphigus disease activity.

## 1. Introduction

Pemphigus is a group of autoimmune bullous diseases that develop due to autoantibodies against desmosomal adhesion molecules (desmoglein (Dsg)1 and Dsg3) in the skin and mucosa.^[1]^ Enzyme-linked immunosorbent assays using recombinant Dsg3 and Dsg1 are widely used to detect circulating autoantibodies. Since disease activity and serum autoantibody titers (index values) are considered parallel, they evaluate response to treatment and disease activity.^[2–5]^ However, it has been reported that anti-Dsg autoantibodies may remain at detectable levels in serum analyses in 17% to 46% of patients in remission without active pemphigus lesions.^[6,7]^ The fact that not all anti-Dsg antibodies are pathogenic explains this situation.^[8]^ Therefore, serum anti-Dsg levels may be misleading when evaluating disease activity.

Immature granulocyte (IG) in peripheral blood indicates increased bone marrow activity. IGs are usually undetected because they are not released into the peripheral blood in healthy people.^[9–11]^ However, they can be detected in peripheral blood when inflammation occurs in the body for any reason. Studies show that IGs are more effective than white blood cells and C-reactive protein (CRP) in determining the severity of inflammation.^[10,12]^ Thanks to technical developments, IG count (IGC) and percentage of IG (IG%) can be easily measured during a routine complete blood count.^[9]^

Pemphigus is a chronic inflammatory skin disease caused by autoimmunity.^[13]^ We aimed to retrospectively evaluate the relationship between the disease activity and IGC, IG%, anti-Dsg1, anti-Dsg3, CRP levels, neutrophil-to-lymphocyte ratios (NLR), and platelet-to-lymphocyte ratio (PLR) in patients with pemphigus.

## 2. Materials and methods

This study is a retrospective, methodological, and valid study.

Pemphigus disease area index (PDAI), IGC and IG% in complete blood count, anti-Dsg1, and anti-Dsg3 serum levels, CRP, NLR, and PLR of patients with pemphigus who were followed between January 2017 and January 2022 in our clinic were recorded retrospectively from the hospital automation system, and the statistical relationship between them was evaluated. Values obtained at times with potential confounding factors that could affect IGC and IG%, such as concomitant infections, use of additional medications other than pemphigus treatment, or other inflammatory conditions, were excluded from the study. Power analysis was used to determine the study sample size. According to the power analysis, the effect size was 1.22 according to the anti-Dsg1 levels during and after treatment in pemphigus patients in the reference study. A total of n: 24 patients were planned to be included in the study in the calculation made by considering alpha 0.05 first type error, beta 0.20-second type error, and 0.80 test power.^[5]^

White blood cell count, neutrophil count, platelet count, lymphocyte count, IGC, and IG% were measured using an automated hematology analyzer (XN 3000; Sysmex Corp., Kobe, Japan). NLR and PLR was calculated manually.

PDAI is a method in which activity (erosion/bullae, new erythema formation; scoring between 0 and 10 according to the number and extent of lesions) and damage (post-inflammatory hyperpigmentation or erythema at the healing lesion site; absent = 0, present = 1) in the skin (in 12 different anatomical areas) and scalp and activity (erosion/bullae; scoring between 0 and 10 according to the number of lesions) in the mucous membranes are evaluated, and total activity scores (between 0 and 250) and total damage scores (between 0 and 13) are obtained.

We certify that we have obtained all necessary permissions from our institution and department to conduct and publish the present work. Approval has been obtained from Kahramanmaraş Sütçü İmam University Noninvasive Clinical Research Ethics Committee, with the decision being dated May 31, 2022 and having no 03, which stated that no obstacles were avoiding the study to be conducted for ethical and scientific aspects. The principles of the Declaration of Helsinki were carried out in our study.

### 2.1. Statistical analysis

SPSS version 22.0 statistical package (SPSS Inc., Chicago, IL) program was used to analyze the data. In the display of the descriptive statistics of the study; mean ± standard deviation, minimum–maximum scores were used for continuous numerical variables, and number (n) and percentage (%) were used for categorical variables. Pearson chi-square and Fisher tests were used to compare categorical variables. According to the normality assessment performed with Kolmogorov–Smirnov and Shapiro–Wilk tests, continuous variables were compared with parametric tests (paired sample *t* test, independent *t* test, ANOVA, and Pearson correlation) where it conformed to normal distribution, and with nonparametric tests (Mann–Whitney *U* test, Kruskal–Wallis test) where it did not conform to normal distribution.

A receiver operating characteristic (ROC) analysis determined the threshold values and sensitivity/specificity of IG% and IGC in predicting disease activity. A multivariate linear regression model was used to identify independent predictors of PDAI. The enter method was used to create the model, and those with significant relationships in the univariate analysis and correlation tests were included in the model. IG% was not added to the model because it has multicollinearity. The model fit was assessed using appropriate residual and goodness-of-fit statistics. The statistical significance level was accepted as *P* < .05.

## 3. Results

A total of 24 patients, 12 women and 12 men, were included in the study. Twenty were patients diagnosed with pemphigus vulgaris (PV), 3 with pemphigus foliaceus, and 1 with pemphigus vegetans. Repetitive intra-patient IGC, IG%, anti-Dsg1 and anti-Dsg3 levels, CRP, NLR and PLR (74 value), and PDAIs (74 PDAI scores) of 24 patients were included in the study.

The average age of the patients was 56.30 ± 16.47.

No statistical correlation was detected between PDAI and anti-Dsg1, anti-Dsg3 serum levels, and NLR (*r* = 0.040, *P* = .737; *r* = 0.007, *P* = .952; *r* = 0.224, *P* = .055, respectively). A statistically significant low correlation was detected between PDAI and CRP (*r* = 0.243, *P* = .037). There was a significant moderate positive correlation between PDAI and IGC, IG%, and PLR (*r* = 0.435, *P* < .001; *r* = 0.412, *P* < .001; *r* = 0.376, *P* = .001, respectively) (Table 1).

**Table 1:** Correlation results between PDAI and other parameters.

	*r*	*P*-value*
PDAI-anti-Dsg 1	0.040	.737
PDAI-anti-Dsg 3	0.007	.952
PDAI-IGC	**0.435**	**<.001**
PDAI-IG%	**0.412**	**<.001**
PDAI-CRP	**0.243**	**.037**
PDAI-NLR	0.224	.055
PDAI-PLR	**0.376**	**.001**

Bold value indicates statistically significant values (*P* < .05).

CRP = C-reactive protein, Dsg = desmoglein, IG% = percentage of immature granulocyte, IGC = immature granulocyte count, NLR = neutrophil-to-lymphocyte ratios, PDAI = pemphigus disease area index, PLR = platelet-to-lymphocyte ratio.

* Pearson correlation test.

**Figure 1.**
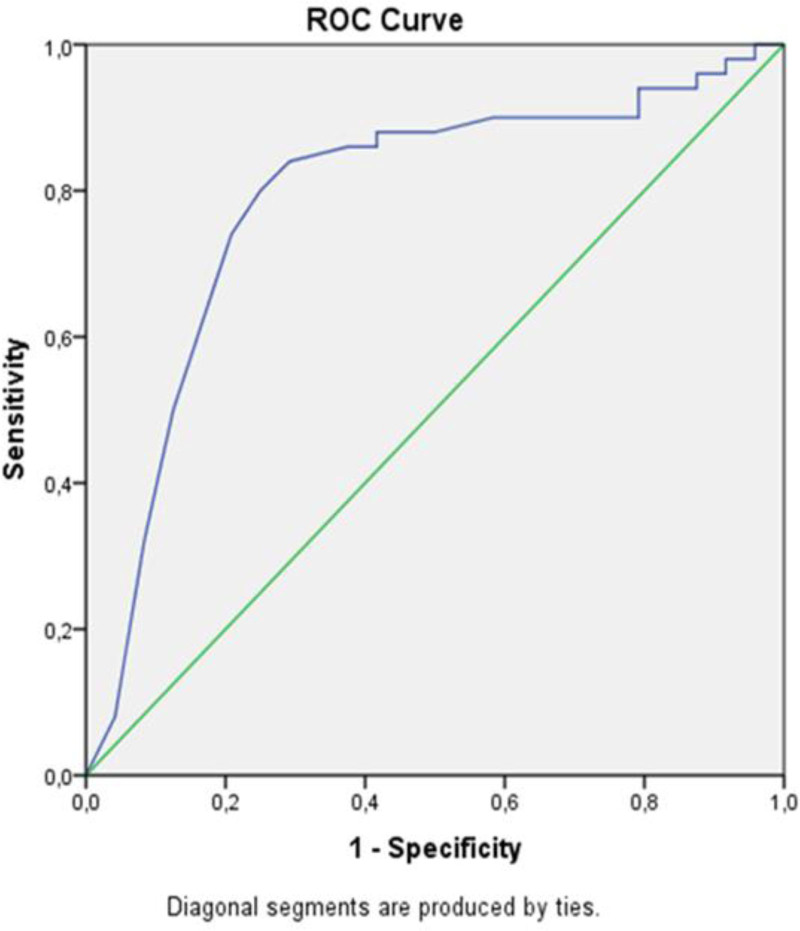
ROC curve analysis of IGC. IGC = immature granulocyte count, ROC = receiver operating characteristic.

ROC curve analysis of IGC was given to show disease severity (Fig. 1 and Table 2). If the IGC cutoff is taken as 0.065, the sensitivity is 0.80, and the specificity is 0.75. Positive predictive value is 92.33% and negative predictive value is 49.92% (AUC: 0.789; CI: 0.67–0.91, *P* < .001).

**Table 2:** Area under the curve: immature granulocyte count.

Area	Standard error^a^	Asymptotic significance^b^	Asymptotic 95% confidence interval	
			Lower bound	Upper bound
0.788	0.060	0.000	0.670	0.905

ROC curve area 0.789 (0.67–0.91), *P* < .001. Sensitivity 80%, specificity 0.75%.

a Signifies standard error of the AUC, indicating the variability of the measurement.

b Signifies asymptotic significance (*P*-value), determining the statistical significance of the test.

Table 3 shows the evaluation of the effects of independent variables in predicting PDAI using multiple linear regression analysis. Accordingly, PLR and the cutoff value of IGC positively predict and significantly affect the PDAI score (*P* < .05). A 1-unit increase in PLR indicates a 0.009 times increase in PDAI, and a 1-unit increase exceeding the IG cutoff value indicates a 2.836 times increase in PDAI (Table 3).

**Table 3:** Evaluation of the effect of independent variables on PDAI score using multiple linear regression analysis.

	β	SE	Standard β	*t*	*P*-value	95% CI	
						Lower	Upper
Anti-Dsg1	0.003	0.004	0.077	0.794	.430	–0.005	0.011
Anti-Dsg3	0.003	0.003	0.077	0.778	.439	–0.004	0.009
IGC_cutoff	2.836	0.664	0.432	4.270	**<.001**	1.510	4.161
CRP	0.014	0.023	0.058	0.609	.544	–0.032	0.060
NLR	–0.059	0.075	–0.119	–0.788	.434	–0.209	0.091
PLR	0.009	0.003	0.475	3.069	**.003**	0.003	0.015

Adjusted R^2^ = 0.384; *F *= 8574; *P* < .001.

Bold value indicates statistically significant values (*P* < .05).

CI = confidence interval, CRP = C-reactive protein, Dsg = desmoglein, IGC = immature granulocyte count, NLR = neutrophil/lymphocyte ratios, PDAI = pemphigus disease area index, PLR = platelet-to-lymphocyte ratio.

## 4. Discussion

Not all anti-Dsg antibodies are pathogenic in patients with pemphigus. The levels of these antibodies may be high in some patients with remission. Furthermore, anti-Dsg autoantibodies may not be detected in the serum of patients with active pemphigus but can be identified in the serum of healthy individuals and those with non-pemphigus diseases. Therefore, anti-Dsg antibody levels may be misleading to indicate pemphigus disease activity.^[8,14,15]^ However, serum anti-Dsg antibody levels are commonly used to assess disease activity in patients with pemphigus, along with the patient’s clinical findings. Therefore the role of anti-Dsg antibody isotypes in assessing disease activity has been investigated. The results of these studies have been inconsistent. Some studies have indicated that IgG4 anti-Dsg3 antibodies are predominant in acute disease, and IgG1 anti-Dsg3 autoantibodies are predominant in chronic disease and remission.^[16–20]^ However, other studies have shown that IgG4 anti-Dsg3 is dominant in remission or that both isotypes are equal at all stages of disease activity.^[21,22]^

Various hematological markers are associated with disease severity in various inflammatory skin diseases (NLR, PLR, and CRP).^[23–26]^ In recent studies, NLR and PLR were determined to decrease remission rates and increase relapse rates in patients with PV. It has been shown that NLR and PLR can monitor response to treatment and detect relapse early.^[27]^ In a study comparing healthy controls and patients with PV, NLR levels were found to be high in patients with PV; it has been shown that there is a moderate correlation between disease severity and anti-Dsg levels. However, no relationship was found between NLR and disease severity, and anti-Dsg levels.^[15]^ In the study of Lin et al, a low/insignificant correlation was found between PDAI and anti-Dsg1 levels, anti-Dsg3 levels, and NLR in patients with PV.^[28]^ According to the results of our study, there was no correlation between PDAI and anti-Dsg1, anti-Dsg3 levels, and NLR; there was a significantly low correlation between PDAI and CRP. However, in the multiple linear regression model, no relationship was observed between CRP and PDAI as an independent variable. A moderate positive correlation was found between PDAI and PLR levels. Furthermore, the results of the multiple linear regression analysis indicated that PLR, when considered as an independent variable, had a significant positive effect on PDAI.

Data obtained from the literature and our results show no definite relationship between PDAI and anti-Dsg1 and anti-Dsg3 levels. Therefore, there is a need for a marker that is easily accessible, inexpensive, and, more sensitive than the aforementioned markers to determine the pemphigus disease activity.

IGs in peripheral blood have recently been introduced as a new inflammatory marker.^[9,12]^ IGs in peripheral blood are an indicator of increased bone marrow activity.^[9,11,12]^ Previous studies have reported that IGC and IG% are more reliable markers than other hematological parameters in diagnosing infection, sepsis, acute appendicitis, internal organ damage in Henoch–Schonlein Purpura, and metastasis in malignancies.^[10,12,29–31]^ In a study, new hematological parameters indicating the activation of immune cells have been investigated in patients with pemphigus; IGC was found to be statistically higher in pemphigus patients compared to healthy controls. The researchers found a significant relationship between increased active lymphocytes and PDAI. However, they didn’t find a significant relationship between IGC and PDAI.^[32]^ Our study found a moderate positive correlation between PDAI and the IGC and IG%. It also shows that the higher IGC cutoff value in the multiple linear regression model is associated with a significant increase in PDAI score. Both ROC analysis and the multiple regression model supported the importance of the IGC cutoff value. A 1-unit increase exceeding the IG cutoff value indicates a 2.836 times increase in PDAI.

By monitoring IGC in patients with pemphigus, the severity of pemphigus may be detected before it gets worse, or the glucocorticoid dosage may be reduced quickly. Thus, the side effects of glucocorticoids may be reduced and the prognosis of patients may be improved.

The limitations of our study were that all of our patients were using glucocorticoids and another immunosuppressive agent, and we could not evaluate the relationship between biomarkers and treatment response. Newly diagnosed patients with pemphigus should be evaluated with prospective cohort studies and the relationship between treatment response and these biomarkers should be tested.

## 5. Conclusion

Anti-Dsg antibody levels may be misleading when indicating disease activity. IGC can be used to assess pemphigus disease activity. Thus, dermatologists can assess the disease activity in patients with pemphigus not only by monitoring the skin lesions and the anti-Dsg levels, but also by using IGC, and plan to treat accordingly. Prospective cohort studies with more patients are required to confirm these results.

## Acknowledgments

We would like to thank Assoc Prof Dr Celal Kuş for his support in the statistical analysis of the study.

## Author contributions

**Conceptualization:** Mine Müjde Kuş, Ozan Can Yaray.

**Data curation:** Ozan Can Yaray.

**Formal analysis:** Mine Müjde Kuş, Ozan Can Yaray.

**Investigation:** Mine Müjde Kuş, Perihan Öztürk, Mehmet Kamil Mülayim.

**Methodology:** Mine Müjde Kuş, Ozan Can Yaray, Perihan Öztürk.

**Resources:** Mine Müjde Kuş, Perihan Öztürk, Mehmet Kamil Mülayim.

**Visualization:** Mine Müjde Kuş, Perihan Öztürk.

**Writing – original draft:** Mine Müjde Kuş, Ozan Can Yaray, Perihan Öztürk.

**Writing – review & editing:** Mine Müjde Kuş, Ozan Can Yaray, Perihan Öztürk, Mehmet Kamil Mülayim.
